# Needle placement errors: do we need steerable needles in interventional radiology?

**DOI:** 10.2147/MDER.S160444

**Published:** 2018-08-03

**Authors:** Tonke L de Jong, Nick J van de Berg, Lisette Tas, Adriaan Moelker, Jenny Dankelman, John J van den Dobbelsteen

**Affiliations:** 1 BioMechanical Engineering Department, Delft University of Technology, Delft, the Netherlands, T.L.deJong@tudelft.nl; 2 Radiology & Nuclear Medicine Department, Erasmus MC, University Medical Center, Rotterdam, the Netherlands

**Keywords:** clinical use, interventional radiology, needle bending, needle deflection, needle placement error, questionnaire, steerable needle

## Abstract

**Purpose:**

Accurate and precise needle placement is of utmost importance in interventional radiology. However, targeting can be challenging due to, eg, tissue motion and deformation. Steerable needles are a possible solution to overcome these challenges. The present work studied the clinical need for steerable needles. We aimed to answer three subquestions: 1) What are the current challenges in needle placement? 2) What are allowable needle placement errors? and 3) Do current needles need improvement and would steerable needles add clinical value?

**Methods:**

A questionnaire was administered at the Annual Meeting of Cardiovascular and Interventional Radiology Society of Europe in 2016. In total, 153 respondents volunteered to fill out the survey, among them 125 (interventional) radiologists with experience in needle placement.

**Results:**

1) Current challenges in needle placement include patient-specific and technical factors. Movement of the target due to breathing makes it most difficult to place a needle (90%). 2) The mean maximal allowable needle placement error in targeted lesions is 2.7 mm. A majority of the respondents (85%) encounter unwanted needle bending upon insertion. The mean maximal encountered unwanted needle bending is 5.3 mm. 3) Needles in interventional radiology need improvement, eg, improved needle visibility and manipulability, according to 95% of the respondents. Added value for steerable needles in current interventions is seen by 93% of the respondents.

**Conclusion:**

Steerable needles have the potential to add clinical value to radiologic interventions. The current data can be used as input for defining clinical design requirements for technical tools, such as steerable needles and navigation models, with the aim to improve needle placement in interventional radiology.

## Introduction

In interventional radiology, needles are placed under image guidance into organs to treat or diagnose patients, eg, in thermal ablation and biopsy procedures, respectively. However, accurate and precise needle placement is challenging, due to several patient-specific and technical factors, such as tissue motion and deformation. Solutions to challenges in needle placement can, eg, be found in developments in imaging, improved instruments and guiding tools, and better training. One proposed technical innovation to overcome some of the challenges in needle placement, and thus decreasing the needle placement error in interventional radiology, is a steerable needle. Such a needle would not only help in decreasing the placement error, but also in reducing the number of punctures and lowering the overall procedure time.

### Background on steerable needles and mechanisms

Several medical engineering research groups are working on the development of steerable needles. According to research, these needles can be used to correct unwanted needle bending and lesion motion,^2^,^3^ actively steer around anatomical obstacles,^2^ or even reach anatomical targets that are not accessible using conventional needles.^4^,^5^

**Figure 1:**
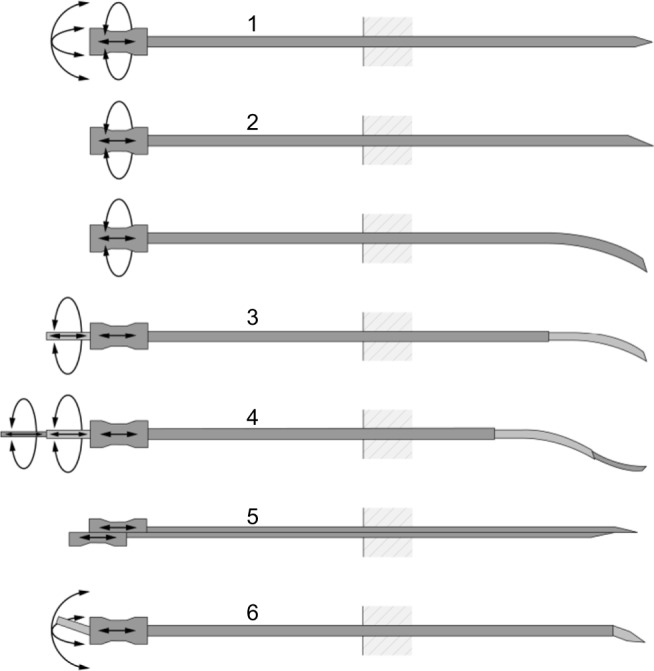
Examples of steerable needles and their degrees of freedom in actuation. **Notes:** The depicted techniques are: 1) base manipulation, 2) bevel tip with and without precurve, 3) precurved stylet, 4) active cannula, 5) programmable bevel, and 6) tendon-actuated tip steering. Picture retrieved from review article on design choices in needle steering. © 2015 IEEE. Reproduced, with permission, from van de Berg NJ, van Gerwen DJ, Dankelman J, van den Dobbelsteen JJ. Design choices in needle steering – a review. *IEEE/ASME T Mech*. 2015;20(5):2172–2183.^1^

An extensive review on design choices in needle steering is given by van de Berg et al.^1^ Figure 1 shows various passive and active needle steering mechanisms. Examples of passive steering mechanisms are manipulation at the base of the needle and asymmetric bevel tip needles. Examples of active mechanisms include precurved needle stylets, active cannulas, programmable bevel tips, and tendon-actuated active needle tips. In addition, an overview of needle-like instruments for steering through solid organs is given by Scali et al.^6^

**Figure 2:**
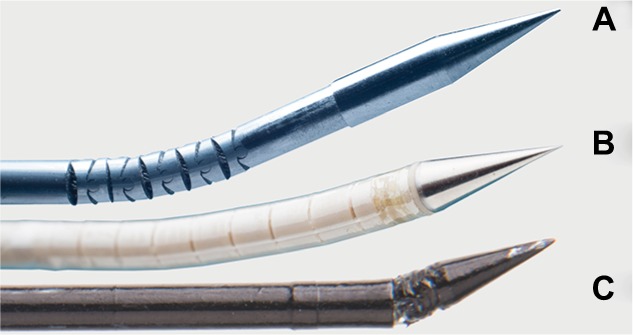
Examples of steerable needle prototypes. **Notes: (A)** A steerable needle with stainless steel segments, **(B)** an MRI-compatible steerable needle, and **(C)** a needle with a steerable tip positioned on top of a miniature ball joint (all prototypes designed and fabricated in the MISIT lab of the Delft University of Technology, the Netherlands). **Abbreviation:** MISIT, minimally invasive surgery and interventional techniques.

Examples of three steerable needle prototypes with tendon-actuated active needle tips are given in Figure 2. Typically, these needles can be manipulated at the base of the needle to change the direction of the needle tip, after which the shaft of the needle follows upon insertion. They range from 0.8 mm to about 3 mm in diameter and have roughly the same diameter as needles that are currently clinically used in interventional radiology (~0.5–2.5 mm). Steerable needle prototypes can either be manually inserted,^7^–^9^ robotically controlled,^10^,^11^ or inserted with a hybrid approach.^12^,^13^

Steerable needles have mostly been tested in experimental settings with the use of a phantom material or animal tissue. For example, Majewicz et al^14^ studied the repeatability of tip-steerable needle insertions in ex vivo and in vivo canine prostate, kidney, and liver tissue. On top of that, some experiments have been performed in a clinical environment. Podder et al^15^ studied the dosimetric benefit of a curvilinear distribution of seeds for low-dose-rate prostate brachytherapy, by inserting the seeds using a smart bevel tip needle into patients. Furthermore, Murphy et al^16^ described the novel use of a curved steerable needle to access symptomatic osseous lesions in the pelvis and sacrum of seven patients.

#### Rationale and goal

Nowadays, steerable needles are technically feasible to make and produce. However, the current general purpose steerable needle may not be the optimal solution, as a result of 1) the wide variety of clinical tasks in which needles are used in interventional radiology, 2) the case-specific level of task difficulty, and 3) the physiological and anatomical variations within and among patients. Instead, specialized instrument designs may have to be developed to aid specific clinical tasks. To develop such clinically relevant technical tools to improve needle placement, we need more insight into the clinical practice, such as the challenges in needle placement, the magnitude of needle placement errors, and the difficulties in interventions. The clinical view of experts on this matter is crucial for defining the proper indications for needle steering in clinical practice, but also for retrieving the right design criteria for these needles.

Therefore, the goal of the current study was to provide insight into the experts’ view on needle placement errors in interventional radiology and their view on the clinical applicability of steerable needles, by means of a questionnaire. The main question that is aimed to be answered is as follows: Is there a clinical need for steerable needles in interventional radiology? This question was divided into three subquestions:

1. What are the challenges in needle placement in interventional radiology?
2. What is the acceptable needle placement error in current clinical practice?
3. Do current needles in interventional radiology need improvement, and when and where would steerable needles add clinical value?

## Methods

### Research tool and respondents

**Figure 3:**
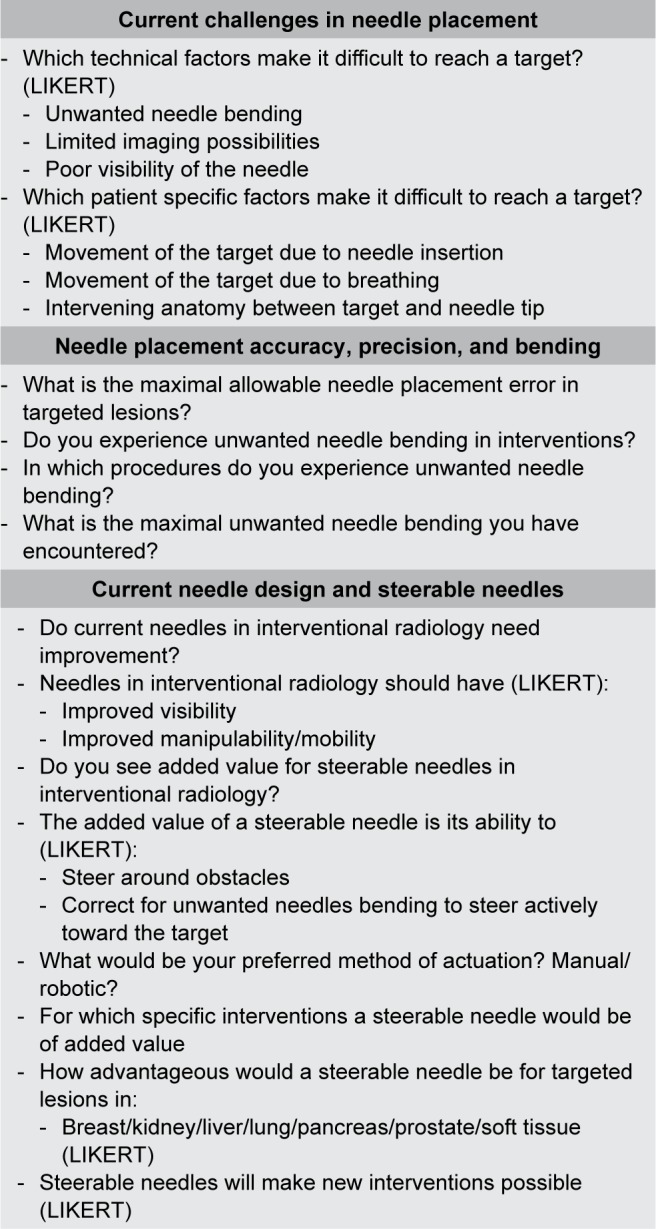
Summarized version of the questionnaire.

A questionnaire was constructed by medical engineers and pretested by interventional radiologists. A summarized version of the questionnaire can be found in Figure 3. The questionnaire was divided in accordance to the subquestions. The section focused on the added value of steerable needles was only filled out by the respondents that shared the opinion that steerable needles would be of added value. All questions were multiple choice, with the ability to add comments, if necessary. Questions regarding the opinion of the respondents were either yes/no questions, or Likert-type (ordinal data) questions with five items.

The questionnaire was conducted at the Annual Meeting of Cardiovascular and Interventional Radiology Society of Europe in 2016, at the technical exhibition. Visitors were asked to fill out the questionnaire and were told that the study investigated the view of clinical experts on needle placement in general and their view on the clinical applicability of steerable needles. In addition, a demonstration of several steerable needle prototypes was given, to familiarize the participants with this concept. Potential participants were approached personally. Data were collected by self-completion of paper questionnaires with the surveyors present.

Review for this research by an institutional review board and written informed consent from the respondents was not required, as we did not record any personal details of the respondents. Furthermore, all data were anonymously processed and archived to ensure privacy of the respondents.

### Data analysis

Response data were analyzed using Matlab 2016b. All (sub) questions were checked for missing data. The percentage of missing data was calculated per question and reported when higher than 20%.

Ordinal Likert-type data are displayed using diverging stacked bar charts. Row counts, ie, the number of radiologists that answered the specific question, are provided for each individual subquestion. Answers were sorted based on the frequencies of positive answers.

Unequal interval data are presented using frequency density histograms with different bin width. Mean values were calculated by multiplying the central x-values of the bin width to the corresponding frequency, after which the summation of these products was divided by the total number of respondents. The mean values were compared among subquestions.

The data from the remaining multiple choice questions regarding clinical interventions are illustrated using bar graphs. Frequencies of the answers to the yes/no questions were calculated and are presented in text.

## Results

### Descriptive statistics

In total, 153 persons filled out the questionnaire voluntarily. A majority of them were (interventional) radiologists with experience in needle placement (n=125, 82%), and were included for further analysis. Other respondents included, but were not limited to, surgeons, medical doctors, and students. The (interventional) radiologists consisted of participants from 40 different countries, with different levels of experience: they all had at least 1 year of experience, whereas 50% of the respondent group had over 10 years of experience.

### Challenges in needle placement

**Figure 4:**
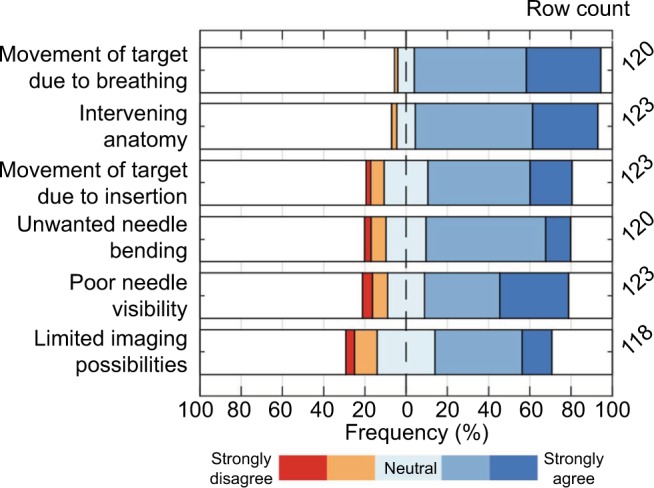
The extent of agreement on: “which factor makes it difficult to reach a target?”

Respondents experience challenges in needle placement in interventional radiology. The overall agreement with each complicating factor is shown in Figure 4. These factors can be divided into patient-specific and technical factors. Examples of patient-specific characteristics that make it difficult to reach a target are movement of the target due to breathing of the patient, intervening anatomy between needle tip and target (eg, ribs and large blood vessels), and movement of the target upon needle insertion. Examples of technical factors are unwanted needle bending/deflection inside tissue, poor needle visibility, and limited imaging possibilities. The figure shows that 90% of the respondents (strongly) agree that the target reachability is complicated by target movements due to breathing, whereas 57% (strongly) agree that the current limits to imaging possibilities play a role. In general, patient-specific factors make it more difficult to reach a target than technical factors, according to the radiologists.

### Needle placement errors

**Figure 5:**
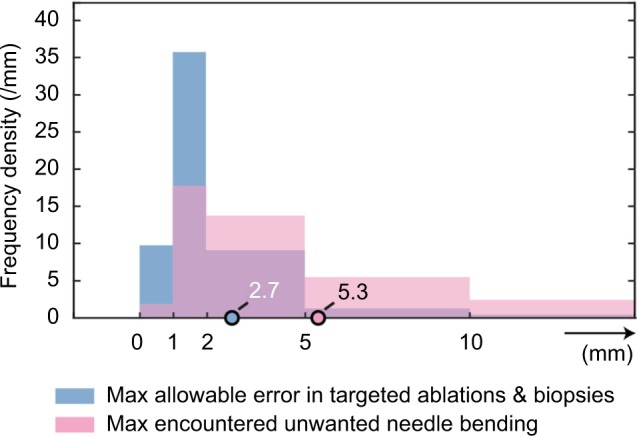
Frequency density distribution of the maximal allowable placement error (mean 2.7 mm) and the maximal encountered unwanted needle bending (mean 5.3 mm).

The aforementioned factors can contribute to needle placement errors, ie, the difference between the needle tip position and its intended position. The respondents were asked to indicate the maximal allowed needle placement error when targeting lesions and to estimate the maximally encountered unwanted needle bending. The results are depicted in Figure 5. Note that three percent of the respondents indicated that zero error is accepted in needle placement. The mean maximal acceptable error was 2.7 mm, as indicated by the circle in the figure. Significant unwanted needle bending in interventions is experienced by 85% of the respondents. The maximal encountered unwanted needle bending is shown in the same figure, by means of the pink bars. The mean maximal encountered unwanted needle bending in interventions was 5.3 mm.

**Figure 6:**
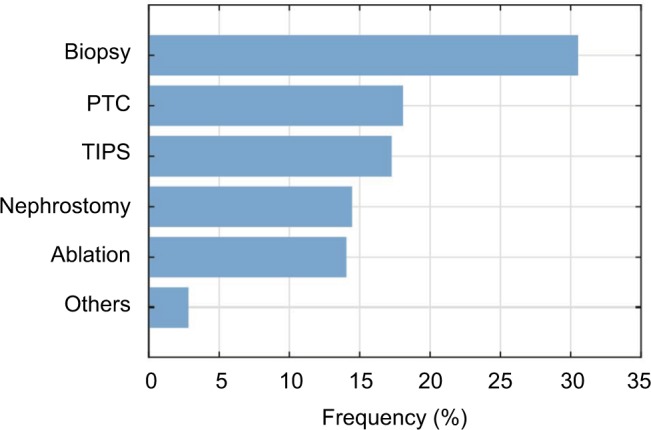
Frequency bar chart of the interventions in which significant unwanted needle bending is encountered (n=125). **Abbreviations:** PTC, percutaneous transhepatic cholangiography; TIPS, transjugular intrahepatic portosystemic shunt.

Figure 6 illustrates the procedures in which the respondents encounter significant needle bending. Biopsies were named the most common ones (>30%), whereas the other procedures were relatively close to each other, and named less frequently.

### Improving needles

**Figure 7:**
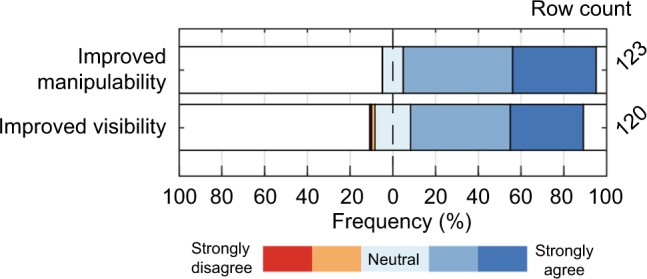
The extent of agreement on “needles in radiology should have improved manipulability and/or improved visibility.”

Most radiologists (95%) share the opinion that current needles in interventional radiology need improvement. The respondents were asked to indicate the extent of their agreement on improvement aspects. In Figure 7, it can be seen that within the respondent group, the desire for an improved needle manipulability/steerability was even larger than the desire for an improved needle visibility. The percentages of the respondents that (strongly) agreed with these desires were 90% and 81%, respectively.

A majority of the respondent group (93%) see added value for steerable needles in interventional radiology. Their preferred actuation method for steerable needles would be manual (91%), with a minority in favor of robotic (9%). However, a preference, here, was indicated by only 42% of the respondents.

**Figure 8:**
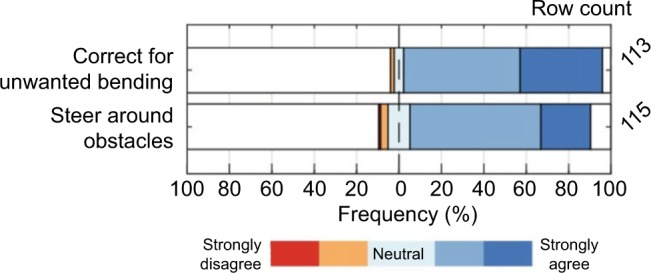
The extent of agreement on “the added value of a steerable needle is its ability to correct for unwanted bending and/or steer around obstacles.”

Figure 8 shows the added value of steerable needles, according the respondent group. The greater number of respondents (94%) agree to some extent that these needles would be helpful to correct for unwanted needle bending to steer actively toward the target, whereas 85% of the respondents agree on this for steering around anatomic obstacles.

**Figure 9:**
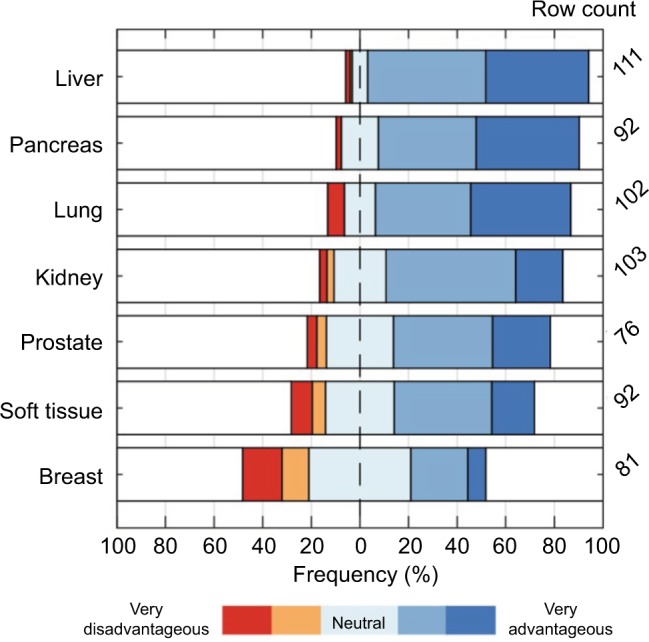
The extent of agreement on “steerable needles would be advantageous for targeted lesions in the specified organs.”

In addition, the respondents were asked to assess the potential benefit of steerable needles for targeted lesions. Results are shown in Figure 9. According to the respondents, a steerable needle would be most advantageous for interventions in the liver (91% advantageous, 3% disadvantageous) and least advantageous for interventions in the breast (31% advantageous, 27% disadvantageous). Missing data of more than 20% were found for the prostate and breast.

**Figure 10:**
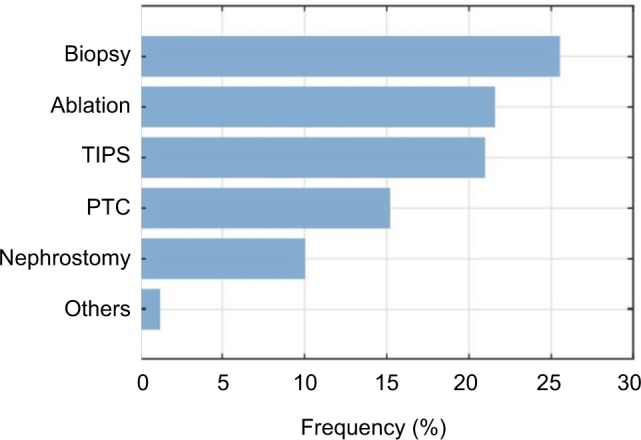
Frequency bar chart of the interventions in which a steerable needle would be of added value (n=125). **Abbreviations:** PTC, percutaneous transhepatic cholangiography; TIPS, transjugular intrahepatic portosystemic shunt.

Figure 10 shows the respondents’ view toward the question: “for which specific interventions would a steerable needle be of added value?” The most frequently named was the biopsy procedure, with 25.5%, whereas nephrostomy and others were named less frequently.

**Figure 11:**
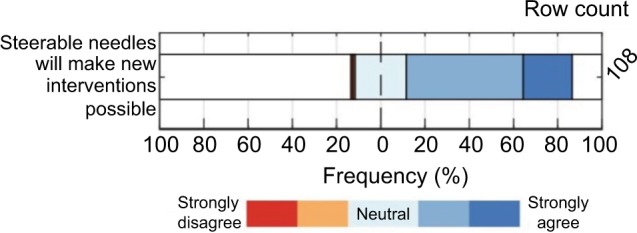
The extent of agreement on “steerable needles would make new interventions possible.”

Finally, respondents were asked to what extent they agreed on the following statement: “steerable needles would make new interventions possible,” and the results are shown in Figure 11. Seventy five percent of the radiologists (strongly) agreed on this, whereas 2% (strongly) disagreed.

## Discussion

This is the first time, to the authors’ knowledge, that a structured research has been carried out on the challenges in needle placement in interventional radiology, and the view of clinical experts on steerable needles, by means of a questionnaire. The present study revealed the most prevalent and important problems in needle placement in interventional radiology.

Limitations of the study should be taken into account when interpreting the results. First, no exact response rate could be given, due to the fact that the questionnaires were manually administered at the Annual Meeting of Cardiovascular and Interventional Radiology Society of Europe. Second, the respondents that were willing to fill out the questionnaire may be biased in favor of new technologies such as steerable needles. However, given the large number of respondents, it is assumed that the presented survey sample gives a representative image of the interventional radiologists’ view.

Although most questions had high completion rates, the percentage of missing data was higher than 20% for the question regarding preferred actuation method for steerable needles. Moreover, the row counts for prostate and breast, as shown in Figure 9, are below 80% (61% and 65%, respectively). Possible explanations could be the low familiarity with the associated interventions and or definitions.

In the current large-scale questionnaire among radiologists, it has been demonstrated that the alleged minimally required accuracy for needle placement is not sufficiently reached in clinical practice. The respondents ranked possible factors that can contribute to needle placement errors. Among respondents, steerable needles were considered a viable alternative to improve current interventions.

It should be noted that the developments in image guidance systems needed for steerable needle imaging were not part of the focus of the presented work. Nonetheless, we stress the importance of reliable and robust imaging systems to be used with steerable needles, as these needles move out-of-plane in conventional two dimensional ultrasound imaging. We believe that 3D ultrasound will be a good solution to this problem, only if the resolution is improved. A recent study showed that needles with arrays of kerfs, often found in compliant joint structures of tip-steered needles, have better contrast-to-noise ratio on ultrasound images than smooth surface needles.^17^ Another solution would be automatic image guidance to keep the needle tip in plane.^18^

The findings of the current study can guide medical engineers in their developments of technical tools to improve needle placement accuracy and precision in clinical practice. More specifically, this will result in improved understanding of the clinical context for engineers to work with and could result in enhanced clinical design requirements for steerable needles and other technical tools in interventional radiology.

## Conclusion

The answers to the questions stated in the “Introduction” section are as follows:

1. Challenges in needle placement in interventional radiology concern patient-specific and technical factors. Remarkably, the respondents found patient-specific factors more of a challenge than the technical factors. For people involved in the development of new medical tools for interventional radiology, the take-home message is therefore to focus not only on improving imaging quality and needle visibility, but also on finding solutions for patient-specific challenges. One could think of steerable needles that can be steered during insertion, but also path planners that incorporate breathing motion of the patient and tissue properties.
2. Significant unwanted needle bending is experienced by the majority of the interventional radiologists (85%). Unwanted needle bending complicates placing the needle at the right spot, induces repuncturing, and thus increases procedure time. The mean maximal encountered unwanted needle bending in interventional procedures is 5.3 mm. However, the mean acceptable needle placement error in targeted lesions is considered as small as 2.7 mm. This implies that unwanted needle bending, which is only one complicating factor in needle placement, is higher than what is considered acceptable.
3. Current needles in interventional radiology need improvement, according to 95% of the (interventional) radiologists. One might think of improved manipulability/steerability, but also improved needle visibility. According to 93% of the respondents, steerable needles would be of added value in interventional radiology. More specifically, most clinical added value can be found for biopsies and ablations in livers. In addition to these conclusions, most of the interventional radiologists foresee that steerable needles not only would add clinical value to current procedures, but also would make new interventions possible.

All in all, we can conclude that steerable needles have the potential to add clinical value to current procedures, with the aim to improve needle placement in interventional radiology.

## References

[1] van de Berg NJ, van Gerwen DJ, Dankelman J, van den Dobbelsteen JJ (2015). Design choices in needle steering – a review. IEEE/ASME T Mech.

[2] Webster RJ, Memisevic J, Okamura AM (2005). Design considerations for robotic needle steering. ICRA 2005.

[3] Kratchman LB, Rahman MM, Saunders JR, Swaney PJ, Webster RJ Toward robotic needle steering in lung biopsy: a tendon-actuated approach.

[4] Cowan NJ, Goldberg K, Chirikjian GS, Rosen J, Hannaford B, Satava RM (2011). Robotic needle steering: design, modeling, planning, and image guidance. Surgical Robotics - Systems, Applications, and Visions.

[5] Reed KB, Majewicz A, Kallem V (2011). Robot-assisted needle steering. IEEE Rob Autom Mag.

[6] Scali M, Pusch TP, Breedveld P, Dodou D (2017). Needle-like instruments for steering through solid organs: A review of the scientific and patent literature. Proc Inst Mech Eng H.

[7] Okazawa S, Ebrahimi R, Chuang J, Salcudean SE, Rohling R (2005). Hand-held steerable needle device. IEEE/ASME Trans Mech.

[8] van de Berg NJ, Dankelman J, van den Dobbelsteen JJ (2017). Endpoint accuracy in manual control of a steerable needle. J Vasc Interv Radiol.

[9] Sears P, Dupont P A steerable needle technology using curved concentric tubes.

[10] Neubach Z, Shoham M (2010). Ultrasound-guided robot for flexible needle steering. IEEE Trans Biomed Eng.

[11] Gilbert HB, Neimat J, Webster RJ (2015). Concentric tube robots as steerable needles: achieving follow-the-leader deployment. IEEE Trans Robot.

[12] Rossa C, Tavakoli M (2017). Issues in closed-loop needle steering. Control Eng Pract.

[13] Rossa C, Usmani N, Sloboda R, Tavakoli M (2017). A hand-held assistant for semiautomated percutaneous needle steering. IEEE Trans Biomed Eng.

[14] Majewicz A, Marra SP, van Vledder MG (2012). Behavior of tip-steerable needles in ex vivo and in vivo tissue. IEEE Trans Biomed Eng.

[15] Podder TK, Dicker AP, Hutapea P, Darvish K, Yu Y (2012). A novel curvilinear approach for prostate seed implantation. Med Phys.

[16] Murphy DT, Korzan JR, Ouellette HA, Liu DM, Clarkson PW, Munk PL (2013). Driven around the bend: novel use of a curved steerable needle. Cardiovasc Intervent Radiol.

[17] van de Berg NJ, Sánchez-Margallo JA, Langø T, van den Dobbelsteen JJ (2018). Compliant joint echogenicity in ultrasound images: towards highly visible steerable needles. Proc SPIE 10576X Medical Imaging.

[18] Kallem V, Cowan NJ (2009). Image guidance of flexible tip-steerable needles. IEEE Trans Robot.

